# Multiple deleted regions on the long arm of chromosome 6 in astrocytic tumours

**DOI:** 10.1054/bjoc.1999.0961

**Published:** 2000-01-18

**Authors:** A Miyakawa, K Ichimura, E E Schmidt, S Varmeh-Ziaie, V P Collins

**Affiliations:** Department of Pathology, Division of Molecular Histopathology, University of Cambridge, Addenbrooke's Hospital, Hills Road, Cambridge, CB2 2QQ, UK; Department of Tumour Pathology, Karolinska Institute, Stockholm, 171 76, Sweden; Department of Urology, Faculty of Medicine, University of Ryukyus, Naha City, Okinawa, 903-0804, Japan; Department of Urology, School of Medicine, Keio University, Tokyo, Japan

**Keywords:** astrocytoma, glioblastoma, microsatellite analysis, allelic loss, tumour progression, clonal heterogeneity

## Abstract

Chromosome 6 deletions are common in human neoplasms including gliomas. In order to study the frequency and identify commonly deleted regions of chromosome 6 in astrocytomas, 159 tumours (106 glioblastomas, 39 anaplastic astrocytomas and 14 astrocytomas malignancy grade II) were analysed using 31 microsatellite markers that span the chromosome. Ninety-five per cent of cases with allelic losses had losses affecting 6q. Allelic losses were infrequent in astrocytomas malignancy grade II (14%) but more usual in anaplastic astrocytomas (38%) and glioblastomas (37%). Evidence for clonal heterogeneity in the astrocytomas and anaplastic astrocytomas was frequently observed (i.e. co-existence of subpopulations with and without chromosome 6 deletions). Clonal heterogeneity was less common in glioblastomas. Five commonly deleted regions were identified on 6q. These observations suggest that a number of tumour suppressor genes are located on 6q and that these genes may be involved in the progression of astrocytic tumours. © 2000 Cancer Research Campaign


British Journal of Cancer (2000) **82**(3), 543–549
© 2000 Cancer Research Campaign
DOI: 10.1054/ bjoc.1999.0961, available online at http://www.idealibrary.com on IDEAL®

### Multiple deleted regions on the long arm of chromosome 6 in astrocytic tumours

**A Miyakawa[1,2,3,4], K Ichimura[1,2], EE Schmidt[1,2], S Varmeh-Ziaie[1,2] and VP Collins[1,2]**

[1]Department of Pathology, Division of Molecular Histopathology, University of Cambridge, Addenbrooke's Hospital, Hills Road, Cambridge CB2 2QQ, UK; [2]Department of Tumour Pathology, Karolinska Institute, Stockholm 171 76, Sweden; [3]Department of Urology, Faculty of Medicine, University of Ryukyus, Naha City, Okinawa 903-0804, Japan; [4]Department of Urology, School of Medicine, Keio University, Tokyo, Japan

**Summary** Chromosome 6 deletions are common in human neoplasms including gliomas. In order to study the frequency and identify commonly deleted regions of chromosome 6 in astrocytomas, 159 tumours (106 glioblastomas, 39 anaplastic astrocytomas and 14 astrocytomas malignancy grade II) were analysed using 31 microsatellite markers that span the chromosome. Ninety-five per cent of cases with allelic losses had losses affecting 6q. Allelic losses were infrequent in astrocytomas malignancy grade II (14%) but more usual in anaplastic astrocytomas (38%) and glioblastomas (37%). Evidence for clonal heterogeneity in the astrocytomas and anaplastic astrocytomas was frequently observed (i.e. co-existence of subpopulations with and without chromosome 6 deletions). Clonal heterogeneity was less common in glioblastomas. Five commonly deleted regions were identified on 6q. These observations suggest that a number of tumour suppressor genes are located on 6q and that these genes may be involved in the progression of astrocytic tumours. © 2000 Cancer Research Campaign

**Keywords:** astrocytoma; glioblastoma; microsatellite analysis; allelic loss; tumour progression; clonal heterogeneity

Karyotypic abnormalities of chromosome 6, especially 6q, represent a frequent cytogenetic alteration associated with a variety of human malignancies (Teyssier and Ferre, 1992). In a compilation of the results from 3185 neoplasms, Mertens et al reported that cytogenetically identified deletions of a part of the long arm of chromosome 6 were commonly observed in 11 types of solid tumours, including malignant melanomas and glioblastomas (Mertens et al, 1997). Other cytogenetic studies have shown involvement of 6q in both low-grade and high-grade astrocytic tumours (Jenkins et al, 1989; Thiel et al, 1992). Comparative genomic hybridization (CGH) analysis and restriction fragment length polymorphism (RFLP) studies have confirmed a high incidence of deletion of 6q in astrocytic gliomas (Fults et al, 1990; Ransom et al, 1992; Liang et al, 1994; Mohapatra et al, 1995; Weber et al, 1996), while allelic loss on 6p is limited (Saitoh et al, 1998). Further evidence for the presence of tumour suppressor gene(s) on chromosome 6 comes from microcell-mediated chromosome transfer into melanoma cell lines that resulted in suppression of metastatic ability and even tumorigenicity (Trent et al, 1990; Welch et al, 1994). Despite all these data no tumour suppressor gene(s) have definitely been identified on chromosome 6.

Deletion mapping of chromosomes 9, 10, 13, and 17 were instrumental in the identification of tumour suppressor genes targeted in the oncogenesis and progression of the adult astrocytic gliomas (Fults et al, 1990; Ransom et al, 1992; Ichimura et al, 1994). Definition of commonly lost regions and the identification of homozygous deletions led to the following genes being implicated in glial oncogenesis or progression: *CDKN2A/B* at 9p21 (Schmidt et al, 1994, 1997), *PTEN/MMAC1* at 10q23 (Li et al, 1997), *RB1* at 13q14 (Henson et al, 1994; Ichimura et al, 1996) and *TP53* at 17p13 (Rasheed et al, 1994).

We report here on an attempt to minimize the region on chromosome 6 that is likely to contain a tumour suppressor gene implicated in adult astrocytic gliomas. The World Health Organization (WHO) classifies these tumours into astrocytomas malignancy grade II, anaplastic astrocytomas malignancy grade III and glioblastomas malignancy grade IV (Kleihues et al, 1993). Progression from less to more malignant forms of these tumours has been well documented – but all may also arise de novo (Russell and Rubinstein, 1989). A series of 159 adult astrocytic gliomas of all three malignancy grades have been deletion mapped by studying 31 loci. A number of commonly deleted regions on the long arm of chromosome 6 have been identified and these deletions have been shown to occur most frequently in the grade III and IV tumours, indicating a role in progression.

### MATERIALS AND METHODS

### Patient material

Fresh surgical specimens from tumour tissues collected at the Karolinska Hospital, Stockholm, and the Sahlgrenska Hospital, Gothenburg, were stored at –135°C for up to 5 years before DNA extraction. Preoperative blood samples from the patients were also collected and stored at –20°C. A portion of each tumour piece frozen was examined histopathologically to estimate the proportion of tumour cells. Pieces containing a high percentage of tumour cells, generally more than 90% and a minimum of 70%, were submitted for analysis. A total of 159 human astrocytic

*Received 13 April 1999*
*Revised 19 July 1999*
*Accepted 25 August 1999*

*Correspondence to:* VP Collins, Department of Pathology, Division of Molecular Histopathology, University of Cambridge, Addenbrooke's Hospital, PO Box 235, Hills Road, Cambridge, CB2 2QQ, UK

tumours including 106 glioblastomas (GB, 13 recurrent tumours), 39 anaplastic astrocytomas (AA, 13 recurrent tumours), 14 astrocytomas grade II (A) were analysed. The study has been approved by the ethical committee of the Karolinska Institute (No. 91:16).

### DNA extraction

DNA extraction was performed as described previously (Ichimura et al, 1996). Briefly, tumour pieces were homogenized in 4 M guanidine thiocyanate solution, overlaid on 5.7 M caesium chloride buffer and centrifuged at 170 000 ***g*** for 16 h at 23°C. DNA was then extracted from the CsCl phase. Blood samples were treated with 5% Triton X-100 in 0.32 M sucrose to isolate nuclear pellets. The nuclear pellets were digested using proteinase K and the DNA was extracted by phenol–chloroform extraction.

### Microsatellite analysis

The following 31 microsatellite markers which span the entire chromosome 6 were used (Figure 1): *D6S344, D6S1574, D6S309, D6S470, D6S1721, D6S429, D6S260, D6S276* and *D6S282* on 6p, *D6S430, D6S460, D6S275, D6S434, D6S1563, D6S1657, D6S407, D6S1572, D6S975, D6S292, D6S311, D6S441, D6S1577, D6S415, D6S437, D6S1581, D6S305, D6S1599, D6S1719, D6S297, D6S1590* and *D6S281* on 6q. All microsatellite markers were purchased from Research Genetics (Huntsville, AL, USA). The data were analysed on the basis of the order of microsatellite markers on the chromosome as reported by the CEPH/Genethon Chromosome 6 linkage map (Figure 1, GDB: 1104325) (Dib et al, 1996). The average distance between each marker was 5.2 cM (the minimum was 1.1 cM and the maximum was 21.5 cM, Figure 1). Paired tumour and blood DNA samples at a concentration of 10 ng $\mu l^{-1}$ were aligned in 96-well plates and polymerase chain reaction (PCR) mixtures were prepared using a Biomek 2000 (Beckman Instruments, Fullerton, CA, USA). The optimal annealing temperature was determined for each primer pair using a RoboCycler Gradient 96 (Stratagene, La Jolla, CA, USA). PCR was performed for each template DNA (40 ng) in 8 μl reaction volumes with 10 mM Tris pH 8.3, 50 mM potassium chloride, 12.5 μM of each dATP, dCTP, dGTP and dTTP, 1.5 mM of magnesium chloride, 0.5 μM of each primer, 0.7–2 μCi of [α-$^{33}$P]dATP and 0.2 U of TaqGold polymerase (Perkin-Elmer, Norwalk, CT, USA). After initial denaturation at 94°C for 15 min, 18–22 cycles of amplification were performed with denaturation at 94°C for 1 min, annealing at the optimized temperature for 1 min, and extension at 72°C for 1 min, followed by a final extension for 10 min on a GeneAmp PCR System 9600 (Perkin-Elmer). The PCR products were electrophoresed on a 5 or 6% non-denaturing polyacrylamide gel, depending on the fragment size, using GeneLoader II (Bio-Rad, Hercules, CA, USA). Gels were transferred to Whatman 3 MM papers, dried and exposed to Storage phosphor screens. The screens were then scanned using a PhosphorImager (Molecular Dynamics, Sunnyvale, CA, USA) and the images were analysed by ImageQuant version 3.3 software (Molecular Dynamics).

### RESULTS

Assessment of the allelic status in tumours was carried out in the following manner. Using ImageQuant software a densitometric profile was drawn across the bands for blood and for tumour. A decrease in signal intensity from one of the alleles in tumour compared to blood was judged as allelic imbalance. In most cases with allelic imbalance, one allele had almost completely disappeared in tumour and this was interpreted as complete allelic loss (Figure 2: GB55 at *D6S415*, GB56 at *D6S1719* and GB140 at *D6S1577*). The residual signals from some tumours were decreased by 30 to 70% of the corresponding normal band in blood (visual assessment of densitometric profiles) (Figure 2: GB8 at *D6S1657*, GB145 at *D6S311*, GB86 at *D6S311*, AA65 at *D6S1719* and AA90 at *D6S305*). In these cases the high residual signal was not attributed to normal cells since the cell content in the tumour pieces was 70% or more (Figure 1). In addition the same tumour could show almost complete allelic loss at some loci and partial allelic loss at others (*D6S434* and *D6S275* in GB47, Figure 3). Such observations were interpreted as the existence of distinct tumour subpopulations with and without deletion at a particular locus within the tumour tissue (Ichimura et al, 1998). This phenomenon was designated as partial allelic loss.

Fifty-six of the 159 tumours (35%) demonstrated allelic loss at one or more loci on chromosome 6 (Tables 1 and 2). These included 39/106 (37%) glioblastomas, 15/39 (38%) anaplastic astrocytomas and 2/14 (14%) astrocytomas. Ten tumours (eight glioblastomas and two anaplastic astrocytomas) showed allelic loss at all informative loci, consistent with monosomy of chromosome 6. Forty-six tumours had allelic loss at one or more informative loci with retention of both alleles at a minimum of one locus (partial deletion of chromosome 6). Among these, three tumours (GB12, GB44 and AA7, Figure 1) had allelic loss only on 6p, while the majority, 34 tumours (24 glioblastomas, nine anaplastic astrocytomas and one astrocytoma) had losses only on 6q. Nine tumours (five glioblastomas, three anaplastic astrocytomas and one astrocytoma) had losses of alleles on both 6p and 6q. In total only 22/159 (14%) tumours had allelic loss at one or more loci on 6p. Allelic loss at one or more 6q loci was demonstrated in a total of 53 tumours (33%; 37 glioblastomas, 14 anaplastic astrocytomas and two astrocytomas) (Table 1). The incidence of allelic loss was almost identical among glioblastomas and anaplastic astrocytomas but considerably lower in astrocytomas.

To determine commonly deleted regions (CDRs), cases showing allelic loss were compared (Figure 1). When a locus

**Table 1** Frequencies and extent of chromosome 6 allelic loss in astrocytic gliomas

| | No. of tumours | Any deletions | Only 6p deletions | Only 6q deletions | 6p and 6q deletions | Monosomy |
|---|---|---|---|---|---|---|
| GB | 106 | 39 (37%) | 2 (2%) | 24 (23%) | 5 (5%) | 8 (8%) |
| AA | 39 | 15 (38%) | 1 (3%) | 9 (23%) | 3 (8%) | 2 (5%) |
| A | 14 | 2 (14%) | 0 | 1 (7%) | 1 (7%) | 0 |
| Total | 159 | 56 (35%) | 3 (2%) | 34 (2%) | 9 (6%) | 10 (6%) |

GB, glioblastomas; AA, anaplastic astrocytomas; A, astrocytomas grade II.

 © 2000 Cancer Research Campaign

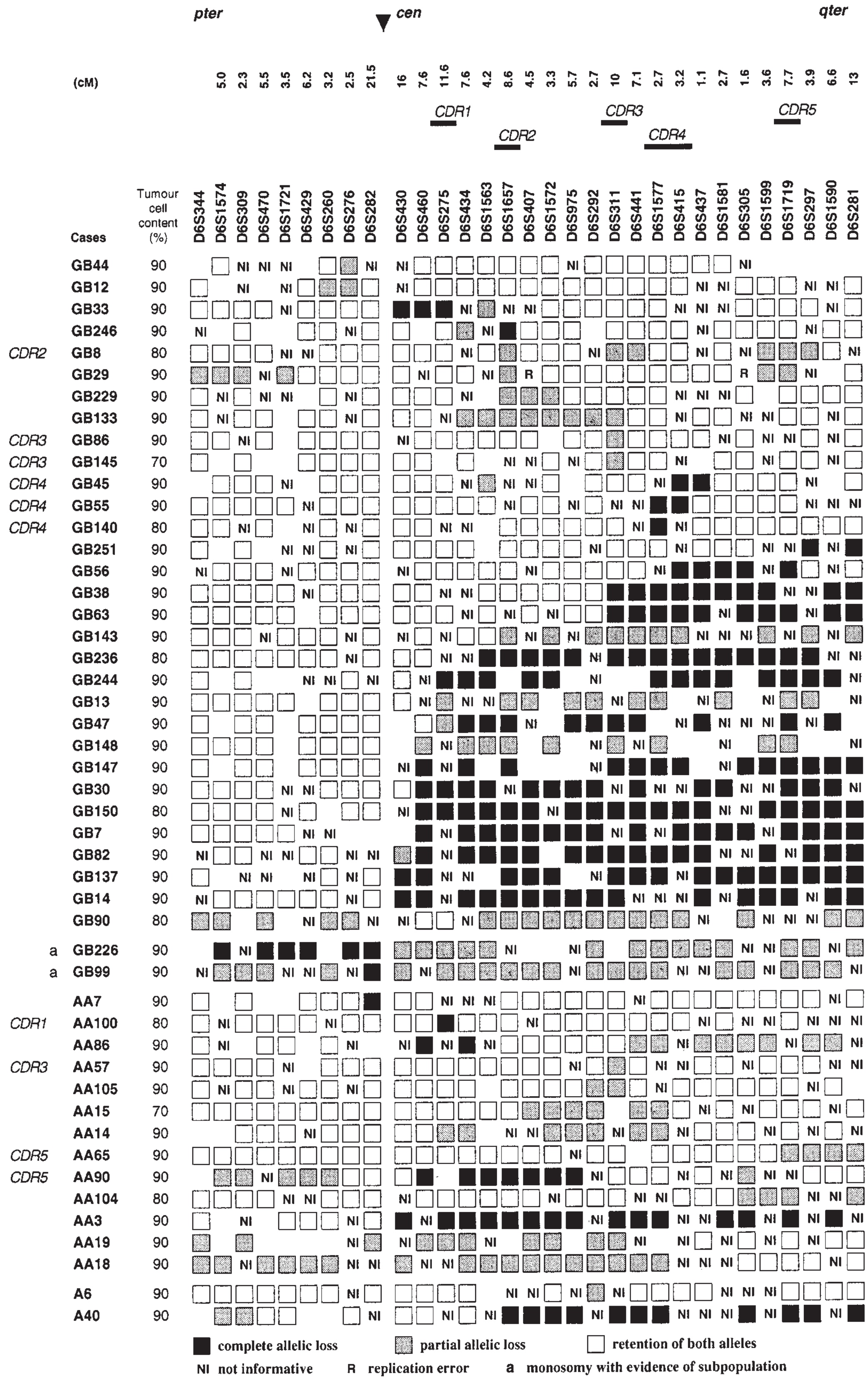

**Figure 1** Deletion mapping of chromosome 6 in human astrocytic tumours showing tumours with loss of alleles. Distances (cM) between each marker according to CEPH/Genethon Chromosome 6 Linkage map (GDB: 1104325) are shown on the top of the panel. The position of the centromere is indicated by an arrow. Cases of monosomy are not shown with the exception of GB226 and GB99. These two cases showed partial loss of alleles at some 6q loci and complete loss at others, indicating subpopulations of tumour cells. Commonly deleted regions (CDRs) are shown as bars above markers (see Results). Cases that define CDRs are marked with the corresponding CDR number on the left

**Table 2** Patterns of allelic loss of chromosome 6 in astrocytic gliomas

| | | Only 6p deletions | Only 6q deletions | 6p and 6q deletions | Monosomy | Total |
|---|---|---|---|---|---|---|
| GB | C | 1 | 13 | 0 | 3 | 17 |
| | P | 1 | 11 | 5 | 5 | 22 |
| AA | C | 1 | 2 | 0 | 1 | 4 |
| | P | 0 | 7 | 3 | 1 | 11 |
| A | C | 0 | 0 | 0 | 0 | 0 |
| | P | 0 | 1 | 1 | 0 | 2 |

GB, glioblastomas; AA, anaplastic astrocytomas; A, astrocytomas grade II; C, complete allelic loss only; P, partial allelic loss, including combination of complete and partial allelic loss.

between a deleted and a non-deleted locus was non-informative, the deletion was considered to extend over the non-informative locus (e.g. *D6S441* and *D6S415* in GB140, Figure 1). When an overlapping region of deletion was defined, it was assumed that the cases share a single critical region. The smallest overlapping deleted region was then defined as the commonly deleted region. Deletions which encompassed more than one minimal overlapping region were not used to define CDRs. According to this principle, five non-overlapping commonly deleted regions (CDR1–5) were defined on 6q. Their sizes ranged from 7 to 19.2 cM (Figure 1).

The most centromeric region of deletion is located between *D6S460* and *D6S434* (CDR1). This 19.2 cM region is defined by an interstitial deletion at *D6S275* with retention of both alleles at *D6S460* and *D6S434* in AA100 (Figure 1). The two bands at *D6S275*

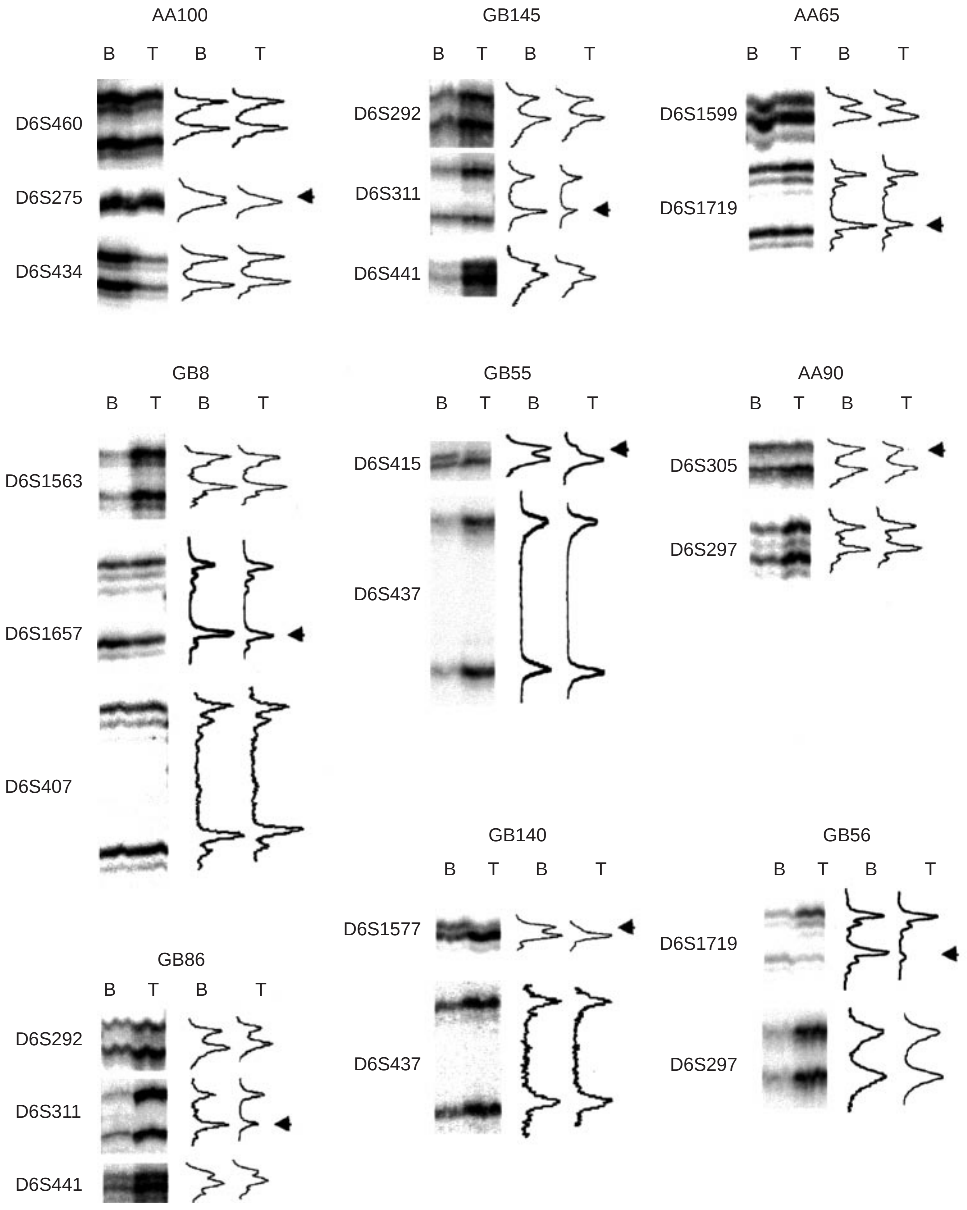

**Figure 2** Examples of microsatellite analysis with densitometric profiles. Cases used to define commonly deleted regions (CDRs) are shown. B, blood; T, tumour. The bands from tumour templates which showed a decrease of signal intensity compared to blood are indicated by an arrow

 *© 2000 Cancer Research Campaign*

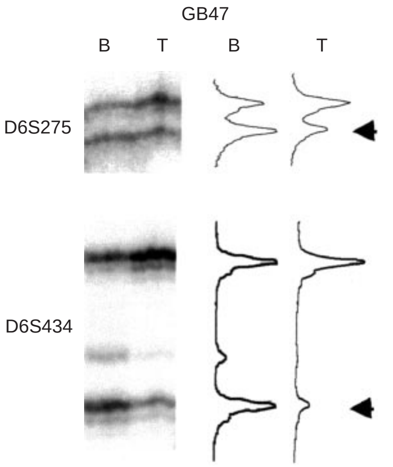

**Figure 3** The same tumour DNA template from GB47 showing partial allelic loss at one locus (*D6S275*) and complete allelic loss at an adjacent locus (*D6S434*). Note the densitometric curve demonstrating partial allelic loss at *D6S275* with approximately 60% decrease of one band and complete allelic loss at *D6S434* with minimum residual signal. The tumour cell content of this case was 90% (see Figure 1)

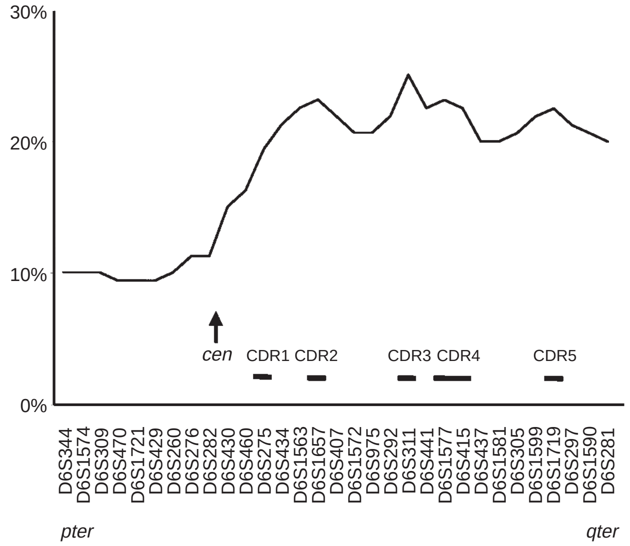

**Figure 4** Incidence of allelic loss at each locus (see text). Defined commonly deleted regions (CDRs) are indicated by horizontal bars. cen: centromere in blood were very close, but it was clear from the densitometric profile that the larger allele was missing in the tumour sample (Figure 2). This region is included in some other small deleted regions in GB33, GB246 and AA86 (Figure 1). Thirty-five tumours (26 glioblastomas and nine anaplastic astrocytomas) showed loss in the CDR1 region.

GB8 had partial allelic loss at *D6S1657* and retained both alleles at *D6S1563* and *D6S407* (Figures 1 and 2). This case defined CDR2, a region of 13.1 cM in size. GB33, GB246, GB29, GB229 and GB45 also had small deletions that include CDR2 (Figure 1). Deleted regions in 39 tumours (29 glioblastomas, nine anaplastic astrocytomas and one astrocytoma) included CDR2.

GB86, GB145 and AA57 showed an interstitial deletion (CDR3, 17 cM) at *D6S311*, flanked by retention at *D6S292* and *D6S441* (Figures 1 and 2). The CDR3 is limited to a 17 cM region by these cases. GB8, AA105 and A6 also had small deletions including CDR3 (Figure 1). Regions with allelic loss in 43 tumours (30 glioblastomas, 11 anaplastic astrocytomas and two astrocytomas) included CDR3.

CDR4 is a 7 cM region between *D6S441* and *D6S437*. The centromeric border of this region is defined by GB45 which had retention of both alleles at *D6S441* and allelic loss at *D6S415* (Figure 1). Allelic loss at *D6S415* (GB55) and *D6S1577* (GB140) with retention of both alleles at *D6S437* defined the telomeric border (Figure 1, 2). Deleted regions in 38 tumours (29 glioblastomas, eight anaplastic astrocytomas and one astrocytoma) involved CDR4.

The most telomeric region (CDR5) lies between *D6S1599* and *D6S297*. The centromeric border for this 11.6 cM region is defined by AA65 which retained both alleles at *D6S1599* and had partial allelic loss at *D6S1719* (Figures 1 and 2). The telomeric border was defined by AA90 which had partial allelic loss at *D6S305* and retention of both alleles at *D6S297* (Figures 1 and 2). The CDR5 was included in the small deletions in GB8, GB29, GB251 and AA104 (Figure 1). Deletions in 36 tumours (28 glioblastomas, seven anaplastic astrocytomas and one astrocytoma) involved CDR5. CDR4 and CDR5 were also included in other larger deletions in GB56, GB38, GB63 and AA86 (Figure 1).

There are six cases which had partial deletions on 6p. Terminal deletions were observed in GB29, AA90 and A40, and interstitial deletions in GB44, GB12 and AA7 (Figure 1). Because of the small number of tumours with deletions, no commonly deleted region was defined.

For the cases with any deletion, partial and complete allelic losses are tabulated (Table 2). Partial loss occurred in two out of two astrocytoma grade II, 11 of 15 (73%) anaplastic astrocytomas and 22 of 39 (56%) glioblastomas.

When the patterns of partial and complete allelic loss are tabulated (Table 2), it appears that partial loss indicating subpopulations of tumour cells within the tumour occur mainly in astrocytomas malignancy grade II (no complete losses found) and anaplastic astrocytomas (the majority (73%) with deletions showed some degree of partial deletion), while 44% of the glioblastomas showed complete losses.

When the overall incidence of loss of alleles along the chromosome is calculated using the rules defined above for the assessment of the size of deletions, we find higher frequencies of loss at the CDRs with a decreased frequencies at 6qter (Figure 4).

The allelic status of chromosome 6 was compared between primary and recurrent tumours. No significant difference was observed.

The tumours used in this study have also been examined for abnormalities of the following tumour suppressor genes and oncogenes: *CDKN2A/p16*$^{INK4A}$, *CDKN2B/p15*$^{INK4B}$, *CDK4*, *MDM2*, *RB1*, *TP53*, *EGFR* and *PTEN/MMAC1* (Ichimura et al, 1996; Liu et al, 1998; Schmidt et al, 1994, 1997, 1999; Ichimura et al, 1999. No significant correlation between chromosome 6 deletion and abnormalities of these genes was identified.

### DISCUSSION

Thirty-three per cent of the cases in our series showed allelic loss at one or more loci on 6q. The incidence is higher than that reported in studies where a smaller number of markers was used

(Fults et al, 1990; Ransom et al, 1992; Liang et al, 1994). The frequency of allelic loss on 6q was similar in glioblastomas (35%) and anaplastic astrocytomas (36%) but much lower in astrocytomas (14%). The observation that the higher malignancy grade tumours showed a higher frequency of allelic loss indicates that a tumour suppressor gene(s) on 6q may play an important role in malignant progression.

We distinguished partial and complete allelic loss. A pattern suggesting partial allelic loss may be observed for several reasons. These include a high percentage of normal cells in the tumour tissue, PCR artefact, allelic gain or the presence of subpopulations in the tumour piece. In this study, only tumour pieces with a high tumour cell content were selected for analysis. For example, GB47 had almost complete loss of one allele at *D6S434*, which indicated a minimal amount of normal cells in the tumour piece. Therefore the high residual signal at *D6S275* was not attributed to normal cells (Figure 3). PCR artefact is unlikely because the extent of signal reduction was reproducible and generally consistent at neighbouring loci. Allelic gain is difficult to distinguish from partial allelic loss by microsatellite analysis alone (Liu et al, 1998). However, it is considered unlikely as CGH studies indicate that abnormalities of chromosome 6 are almost always deletions (Mohapatra et al, 1995; Weber et al, 1996). For these reasons we favour the last possibility – the presence of subpopulations/clones of tumour cells with different allelic status. When a deletion gives a growth/survival advantage, the clone will expand, becoming dominant. The observed partial allelic loss may be a phenomenon which represents this transition. Among cases with allelic loss 2/2 astrocytomas and 11/15 (73%) anaplastic astrocytomas showed evidence of partial allelic loss. The incidence was lower in the glioblastomas; 56% (22/39) of cases with allelic loss have partial allelic loss (Table 2). All these observations support the idea that deletion of tumour suppressor gene(s) on 6q plays an important role in glioma progression.

We have defined five commonly deleted regions. These regions were deduced under the principle described in the Results section. The markers which defined all small deleted regions have been confirmed by at least two independent experiments. The incidences of deletions were relatively similar in CDR2–5 and when graphed, show peaks as compared to adjacent loci (Figure 4). It is also notable that the incidence of allele losses decreased at 6qter arguing against a simple loss of the terminal part of the 6q arm.

Some candidate tumour suppressor genes identified in other tumours fall into the CDRs determined in our study. The Histone deacetylase 2 gene (*HDAC2*) was recently mapped to 6q21 (Betz et al, 1998) between *D6S1563* and *D6S1657*, the CDR2 region. *HDACs* are considered tumour suppressor gene candidates as they may suppress transcription by modulating chromatin structure. The oestrogen receptor gene *ESR* is located between *D6S311* and *D6S441*, the region included in CDR3. While the involvement of *ESR* in gliomas is difficult to support theoretically, its mutation has been reported in a human breast cancer cell line (Ponglikitmongkol et al, 1988). The *LOT-1* (Lost on transformation 1) gene also falls into CDR3 and frequent loss of one copy has been observed in ovarian cancer (Abdollahi et al, 1997). The protein product of *LOT-1* contains zinc finger motifs and is believed to act as a transcription factor. Two candidate senescence genes have been mapped to 6q in the CDR1 and CDR5 regions; *SEN 6A* (Sandhu et al, 1996) between *D6S460* and *D6S434* (CDR1) and *SEN6* (Banga et al, 1997) between *D6S1719* and *D6S281* (CDR5). In glioma, no candidate tumour suppressor genes have ever been proposed on chromosome 6.

Even though some of the CDRs are represented by single markers, the regions defined by our study span large genomic regions. Further narrowing down of the minimum region of deletion is necessary to facilitate focusing on candidate genes to investigate their relevancy as tumour suppressor genes. The ever increasing chromosome and gene data being produced, together with the efforts to localize candidate regions as presented here, facilitate the identification of novel tumour suppressor genes. While it might seem unlikely that all five CDRs represent truly interesting areas the findings here clearly warrant further investigation.

In conclusion, we have confirmed the high frequency of deletion of the long arm of chromosome 6 in malignant astrocytic tumours and have defined a number of critical regions. Clonal heterogeneity was observed but found to diminish in the most malignant tumour group, the glioblastomas. The evidence suggests that the long arm of chromosome 6 harbours tumour suppressor genes which may be involved in malignant progression of astrocytic gliomas.

### ACKNOWLEDGEMENTS

This work was supported by grants from the Swedish Cancer Society, Stockholm's Cancer Society, King Gustaf V Jubilee Fund, Axel and Margaret Ax:son Johnsons Fund, and the Funds of the Karolinska Institute. AM was supported by grants from the Japan–Sweden Foundation, the Canon Foundation and the Uehara Memorial Foundation.

### REFERENCES

Abdollahi A, Roberts D, Godwin AK, Schultz DC, Sonoda G, Testa JR and Hamilton TC (1997) Identification of a zinc-finger gene at 6q25: a chromosomal region implicated in development of many solid tumors. *Oncogene* **14**: 1973–1979

Banga SS, Kim S, Hubbard K, Dasgupta T, Jha KK, Patsalis P, Hauptschein R, Gamberi B, Dalla-Favera R, Kraemer P and Ozer HL (1997) SEN6, a locus for SV40-mediated immortalization of human cells, maps to 6q26–27. *Oncogene* **14**: 313–321

Betz R, Gray SG, Ekström C, Larsson C and Ekström TJ (1998) Human histone deacetylase 2, HDAC2 (human RPD3), is localized to 6q21 by radiation hybrid mapping. *Genomics* **52**: 245–246

Dib C, Faure S, Fizames C, Samson D, Drouot N, Vignal A, Millasseau P, Marc S, Hazan J, Seboun E, Lathrop M, Gyapay G, Morissette J and Weissenbach J (1996) A comprehensive genetic map of the human genome based on 5,264 microsatellites. *Nature* **380**: 152–154

Fults D, Pedone CA, Thomas GA and White R (1990) Allelotype of human malignant astrocytoma. *Cancer Res* **50**: 5784–5789

Henson JW, Schnitker BL, Correa KM, von Deimling A, Fassbender F, Xu HJ, Benedict WF, Yandell DW and Louis DN (1994) The retinoblastoma gene is involved in malignant progression of astrocytomas. *Ann Neurol* **36**: 714–721

Ichimura K, Schmidt EE, Yamaguchi N, James CD and Collins VP (1994) A common region of homozygous deletion in malignant human gliomas lies between the IFN-α/ω gene cluster and the D9S171 locus. *Cancer Res* **54**: 3127–3130

Ichimura K, Schmidt EE, Goike HM and Collins VP (1996) Human glioblastomas with no alterations of the CDKN2A (p16INK4A, MTS1) and CDK4 genes have frequent mutations of the retinoblastoma gene. *Oncogene* **13**: 1065–1072

Ichimura K, Schmidt EE, Miyakawa A, Goike HM and Collins VP (1998) Distinct patterns of deletion on 10p and 10q suggest involvement of multiple tumor suppressor genes in the development of astrocytic gliomas of different malignancy grades. *Genes Chromosomes Cancer* **22**: 9–15

 © 2000 Cancer Research Campaign

Ichimura K, Bolin MB, Goike HM, Schmidt EE, Moshret A and Collins VP (1999) Deregulation of the p14$^{ARF}$/MDM2/p53 pathway is a prerequisite for human astrocytic gliomas with $G_{1/s}$ transition control gene abnormalities. *Cancer Res* (in press)

Jenkins RB, Kimmel DW, Moertel CA, Schultz CG, Scheithauer BW, Kelly PJ and Dewald GW (1989) A cytogenetic study of 53 human gliomas. *Cancer Genet Cytogenet* **39**: 253–279

Kleihues P, Burger PC and Scheithauer BW (1993) *Histological Typing of Tumors of the Central Nervous System*. World Health Organization. International histological classification of tumors. Springer-Verlag: New York

Li J, Yen C, Liaw D, Podsypanina K, Bose S, Wang SI, Puc J, Miliaresis C, Rodgers L, McCombie R, Bigner SH, Giovanella BC, Ittmann M, Tycko B, Hibshoosh H, Wigler MH and Parsons R (1997) PTEN, a putative protein tyrosine phosphatase gene mutated in human brain, breast, and prostate cancer. *Science* **275**: 1943–1947

Liang BC, Ross DA, Greenberg HS, Meltzer PS and Trent JM (1994) Evidence of allelic imbalance of chromosome 6 in human astrocytomas. *Neurology* **44**: 533–536

Liu L, Ichimura K, Pettersson EH and Collins VP (1998) Chromosome 7 rearrangements in glioblastomas; loci adjacent to EGFR are independently amplified. *J Neuropathol Exp Neurol* **57**: 1138–1145

Mertens F, Johansson B, Hoglund M and Mitelman F (1997) Chromosomal imbalance maps of malignant solid tumors: a cytogenetic survey of 3185 neoplasms. *Cancer Res* **57**: 2765–2780

Mohapatra G, Kim DH and Feuerstein BG (1995) Detection of multiple gains and losses of genetic material in ten glioma cell lines by comparative genomic hybridization. *Genes Chromosomes Cancer* **13**: 86–93

Ponglikitmongkol M, Green S and Chambon P (1988) Genomic organization of the human oestrogen receptor gene. *EMBO J* **7**: 3385–3388

Ransom DT, Ritland SR, Moertel CA, Dahl RJ, O'Fallon JR, Scheithauer BW, Kimmel DW, Kelly PJ, Olopade OI, Diaz MO and et al (1992) Correlation of cytogenetic analysis and loss of heterozygosity studies in human diffuse astrocytomas and mixed oligo-astrocytomas. *Genes Chromosomes Cancer* **5**: 357–374

Rasheed BK, McLendon RE, Herndon JE, Friedman HS, Friedman AH, Bigner DD and Bigner SH (1994) Alterations of the TP53 gene in human gliomas. *Cancer Res* **54**: 1324–1330

Russell DS and Rubinstein LJ (1989). Tumours of central neuroepithelial origin. In: *Pathology of Tumours of the Nervous System*, pp. 83–350. Edward Arnold: London.

Saitoh Y, Bruner JM, Levin VA and Kyritsis AP (1998) Identification of allelic loss on chromosome arm 6p in human astrocytomas by arbitrarily primed polymerase chain reaction. *Genes Chromosomes Cancer* **22**: 165–170

Sandhu AK, Kaur GP, Reddy DE, Rane NS and Athwal RS (1996) A gene on 6q 14–21 restores senescence to immortal ovarian tumor cells. *Oncogene* **12**: 247–252

Schmidt EE, Ichimura K, Reifenberger G and Collins VP (1994) CDKN2 (p16/MTS1) gene deletion or CDK4 amplification occurs in the majority of glioblastomas. *Cancer Res* **54**: 6321–6324

Schmidt EE, Ichimura K, Messerle KR, Goike HM and Collins VP (1997) Infrequent methylation of *CDKN2A* (*MTS1/p16*) and rare mutation of both *CDKN2A* and *CDKN2B* (*MTS2/p15*) in primary astrocytic tumours. *Br J Cancer* **75**: 2–8

Schmidt EE, Ichimura K, Goike HM, Moshret A, Liu L and Collins VP (1999) Mutational profile of the *PTEN* gene in primary human astrocytic tumours and cultivated xenografts. *J Neuropathol Exp Neurol* **58**: 1170–1183

Teyssier JR and Ferre D (1992) Identification of a clustering of chromosomal breakpoints in the analysis of 203 human primary solid tumor non specific karyotypic rearrangements. *Anticancer Res* **12**: 997–1004

Thiel G, Losanowa T, Kintzel D, Nisch G, Martin H, Vorpahl K and Witkowski R (1992) Karyotypes in 90 human gliomas. *Cancer Genet Cytogenet* **58**: 109–120

Trent JM, Stanbridge EJ, McBride HL, Meese EU, Casey G, Araujo DE, Witkowski CM and Nagle RB (1990) Tumorigenicity in human melanoma cell lines controlled by introduction of human chromosome 6. *Science* **247**: 568–571

Weber RG, Sommer C, Albert FK, Kiessling M and Cremer T (1996) Clinically distinct subgroups of glioblastoma multiforme studied by comparative genomic hybridization. *Lab Invest* **74**: 108–119

Welch DR, Chen P, Miele ME, McGary CT, Bower JM, Stanbridge EJ and Weissman BE (1994) Microcell-mediated transfer of chromosome 6 into metastatic human C8161 melanoma cells suppresses metastasis but does not inhibit tumorigenicity. *Oncogene* **9**: 255–262

© 2000 Cancer Research Campaign
